# Evaluating the Effects of Sensorimotor Training on the Physical Capacities of Older People

**DOI:** 10.3390/ejihpe15040050

**Published:** 2025-04-01

**Authors:** Carolina A. Cabo, Víctor Hernández-Beltrán, Orlando Fernandes, Cláudia Mendes, José M. Gamonales, Mário C. Espada, José A. Parraca

**Affiliations:** 1 Departamento de Desporto e Saúde, Escola de Saúde e Desenvolvimento Humano, Universidade de Évora, Largo dos Colegiais 2, 7000-645 Évora, Portugal; orlandoj@uevora.pt (O.F.); klaudinha@icloud.com (C.M.); jparraca@uevora.pt (J.A.P.); 2 Comprehensive Health Research Centre (CHRC), University of Évora, Largo dos Colegiais 2, 7000-645 Évora, Portugal; mario.espada@ese.ips.pt; 3 Instituto Politécnico de Setúbal, Escola Superior de Educação, 2914-504 Setúbal, Portugal; 4 Sport Physical Activity and Health Research & Innovation Center (SPRINT), 2040-413 Rio Maior, Portugal; 5 Optimization of Training and Sports Performance Research Group, Faculty of Sport Science, University of Extremadura, 10005 Cáceres, Spain; vhernandpw@alumnos.unex.es (V.H.-B.); martingamonales@unex.es (J.M.G.); 6 CBIOS-Universidade Lusófona’s Research Center for Biosciences and Health Technologies, 1749-024 Lisbon, Portugal; 7 Faculty of Education and Psychology, University of Extremadura, 06006 Badajoz, Spain; 8 Life Quality Research Centre (CIEQV-Setúbal), Instituto Politécnico de Setúbal, 2914-504 Setúbal, Portugal; 9 Centro Interdisciplinar para o Estudo da Performance Humana—CIPER, Faculdade de Motricidade Humana, Universidade de Lisboa, 1499-002 Lisboa, Portugal

**Keywords:** aging, agility, flexibility, strength, physical activity, functional capacity

## Abstract

Background: Physical activity (PA) plays a crucial role in improving the quality of life (QoL) in older people, particularly by enhancing their balance and movement coordination. Objective: This study aimed to assess the effects of sensorimotor training intervention in older adults. Methods: A total of 90 participants, divided into a Control Group (*n* = 44) and Experimental Group (*n* = 46) were involved in a 24-week sensorimotor training program. The physical capacities of the participants were assessed both before and after the intervention program. Strength and flexibility were measured using the “Rikli and Jones” protocol (1999), while agility and speed were assessed through “Timed-up-and-go” tests. Taking into account the participants’ gender, a descriptive analysis of the sample was conducted to describe the data using the mean and standard deviation. Student’s T test was performed to compare the differences between the groups according to the first and second data collection moments (before and after the intervention). Jamovi software (v. 2.5.2.0) was used to develop the statistical analysis, using a *p*-value of less than 0.05 to assess the statistical significance. Results: The Experimental Group showed significant improvements across all the analyzed variables following the intervention (*p* < 0.005), indicating substantial gains in physical capacities. In contrast, the Control Group in the “sitting and reaching” test did not show a significant difference between the groups highlighting the lack of improvement without intervention. According to the effect size of the sample, it was observed that the parameters “reach behind your back (right)” and “reach behind your back (left)” showed the highest effect size comparing the Control Group and Experimental Group (ES: 0.60, 0.71). Conclusions: The findings highlight the practical clinical impact of implementing tailored physical activity programs for older adults. Such interventions are critical for enhancing QoL, reducing the risk of falls, injuries, and chronic illnesses, and promoting overall health, independence, and well-being. Integrating sensorimotor training into the routine care for older people can support healthy aging and functional independence.

## 1. Introduction

Physical activity (PA) is crucial for improving the QoL of older adults, enhancing balance, coordination (Choi et al., 2021; Dunsky, 2019; Prasad et al., 2021), physical and mental health, autonomy, and pain management (Vagetti et al., 2014). Regular PA benefits cardiovascular health, reduces anxiety and depression (Goodarzi et al., 2024), strengthens muscles, improves mobility, and fosters independence (Pfeifer et al., 2022). It also enhances emotional resilience and social interaction, reducing loneliness and increasing life satisfaction (Lindsay Smith et al., 2017).

Conversely, inactivity contributes to weakness, negative health outcomes (Ghram et al., 2021) and higher risks of mortality and cardiovascular diseases (Fletcher et al., 2018; Rizwan Sajid et al., 2021). Modern lifestyles encourage sedentarism (Woessner et al., 2021), with men generally exhibiting better physical function and QoL than women (Prasad et al., 2021). To counteract this, PA promotion programs should be frequent to improve mental health (Cabo et al., 2024) and reduce cardiovascular risks (Ciumărnean et al., 2021).

PA programs for older adults should emphasize balance, coordination, and strength to prevent falls and enhance movement control (Nikitas et al., 2022). Engaging in at least 20 min of daily PA (Fabris & Sinagra, 2022), or 150 min per week of moderate-intensity exercise provides cardiovascular benefits, reduces chronic disease risks, and supports cognitive health (McGarrigle et al., 2020). Such programs also improve mental health (Musdalifah Ahmad, 2023), well-being (Carvalho et al., 2023), and the QoL of older people (Fiorilli et al., 2022).

Physical capacities refer to the fundamental attributes that determine an individual’s ability to perform physical tasks efficiently and safely. These capacities include strength, endurance, flexibility, balance, coordination, and agility, all of which are essential for maintaining mobility, preventing falls, and ensuring the overall functional independence in older adults (Mueller et al., 2021). Strength, particularly in the lower and upper limbs, supports daily activities such as standing up, walking, and carrying objects (Parraca et al., 2022). Endurance allows sustained physical effort, reducing fatigue and improving cardiovascular health (McGarrigle et al., 2020). Flexibility contributes to joint mobility and reduces stiffness, facilitating smoother movements (Shirazi et al., 2023). Balance and coordination enhance postural stability and minimize the risk of falls (Nikitas et al., 2022), while agility ensures quick and efficient movement responses to environmental challenges (Pfeifer et al., 2022).

Assessing and improving these physical capacities is crucial for promoting independence, preventing injuries, and enhancing QoL in aging populations. By evaluating these attributes through standardized tests—such as the Timed Up and Go (TUG) test for mobility, sit and reach test for flexibility, and chair stand test for strength—health professionals can design targeted interventions, like sensorimotor training, to address age-related declines and optimize physical function (Carvalho et al., 2023; Wróblewska et al., 2023).

Assessing the functional capacity helps tailor healthcare strategies and prevent falls (Wróblewska et al., 2023). It should consider mental, physical, and functional dimensions (Wróblewska et al., 2023) using tests like the “Timed Up and Go” (TUG) for mobility (Donisi et al., 2020) and short-term mortality risk (Chua et al., 2020). In this line, muscular endurance and upper and lower limb strength are essential to developing daily routines and activities (Parraca et al., 2022), and this information can be monitored using an isokinetic dynamometer (Li et al., 2006), through the evaluation of torque (Perrin et al., 1987) and power (Janssen & Le-Ngoc, 2009) to determine the lower limb strength. Strength tests, such as the “Sit-to-stand” (Takai et al., 2009) or “chair stand test” (Cho et al., 2012), assess the fall risk, while flexibility can be measured via the “sit and reach” test for the lower limbs (Shirazi et al., 2023; Teraoka et al., 2022) and the “behind the back reach” test for the upper limbs (Correa et al., 2022; de Oliveira et al., 2024). Evaluating and analyzing strength and flexibility help to improve the motor abilities and functional capacity of older people, and in general, their QoL (Melchiorri et al., 2022).

Sensorimotor training integrates sensory input with motor responses to enhance balance, coordination, and functional movement, making it a key strategy in maintaining independence and reducing the fall risk in older adults (Asan et al., 2022; M. Martinez et al., 2009). Aging leads to sensory and motor deterioration, affecting proprioception, muscle strength, and balance control, increasing the fall risk and mobility impairments (Nikitas et al., 2022). This training helps counteract these declines by improving proprioception, neuromuscular coordination, and postural control, while also enhancing the reaction times and adaptability to environmental challenges (Ghram et al., 2021).

Sensorimotor training is widely used in rehabilitation, fall prevention, and physical activity programs for older individuals. It includes exercises that challenge balance, coordination, and sensory integration, such as balance training (e.g., tandem walking, single-leg stance), proprioceptive exercises (e.g., weight-shifting tasks), strength and stability exercises, and dual-task training that combines cognitive and motor tasks (Fabris & Sinagra, 2022; Mueller et al., 2021). These exercises often involve unstable surfaces like balance boards and foam pads or additional sensory challenges like visual occlusion.

Studies show that sensorimotor training improves functional mobility, reduces the fall risk, and enhances the overall physical performance (Carvalho et al., 2023). By incorporating sensorimotor training into structured programs, older individuals can improve their movement efficiency, maintain their independence, and sustain a higher QoL (Cabo et al., 2024).

In short, PA is one of the fundamental pillars for improving the functional capacity and QoL of older people (Cabo et al., 2024). Several health benefits are associated with physical exercise, making it essential to improve our approach to evaluate and control physical activities. This is necessary to ensure that practitioners can continue their activities, minimizing the risk of injuries, dropouts, or lack of motivation (Warburton & Bredin, 2017). Hence, this study aimed to address a gap in the research by assessing various physical abilities in a group of older individuals before and after a sensorimotor training intervention. The study hypothesis was defined as follows: Is sensorimotor training effective in improving the physical abilities of older people? To test and answer the hypothesis, the effects of the intervention on the participants’ physical capacities and performance were evaluated.

## 2. Materials and Methods

### 2.1. Design

A parallel-group randomized controlled trial was conducted, including a 6-month intervention phase and a 1-year follow-up period. For both groups (Control and Experimental), two evaluation moments were carried out, a first moment at the baseline (before starting the intervention for two months, three times a week, for 6 h a day) and after the intervention period, for two months (three times a week, for 6 h a day) (Montero & León, 2007). The Control Group (CG) did not perform any type of activity or physical program throughout the intervention period.

### 2.2. Participants

The study analyzed 90 participants aged between 55 and 80 years old, divided into two groups: the CG (73.70 ± 6.88 years) and the Experimental Group (EG) (72.40 ± 6.88 years). Table 1 shows the characteristics of the CG and EG to contextualize and create a framework for the study sample.

The participants had to meet the following inclusion criteria: (1) age between 55 and 80 years old; (2) without prostheses (except dental prostheses); and (3) have not been involved in surgical intervention 6 months before the study. The exclusion criteria were as follows: (1) musculoskeletal diagnosis; (2) problems in locomotion; (3) psychiatric diseases and neurological disorders; and (4) clinical cardiovascular diagnosis. To establish the inclusion and exclusion criteria, we conducted a thorough anamnesis that included questions regarding the participants’ clinical status.

### 2.3. Ethics

This project was approved by the University of Évora’s Ethics Committee (approval number: 21040; registration number: NCT05398354; https://www.clinicaltrials.gov/ct2/show/NCT05398354?term=NCT05398354&draw=2&rank=1), accessed on 20 January 2025. The study was registered with the Clinical Trials.gov PRS Protocol Registration and Results System. Each participant provided informed consent before participating, according to the Helsinki Declaration for Human Studies (2013).

### 2.4. Intervention

**Figure 1:**
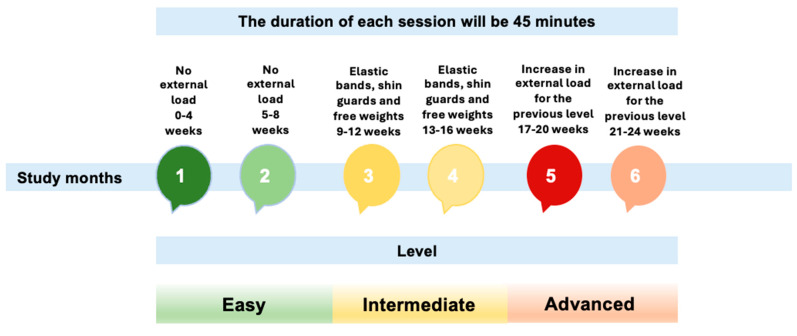
Study timeline graph.

The sensorimotor training program was conducted for 6 months, with a frequency of twice a week. The duration of the sessions was 45 min in total. The training volume consisted of 8 exercises, where each participant performed the maximum number of repetitions within 50 s, completing 4 sets. As the program progressed, the load progressively increased. To achieve this, the session was divided into three levels of intensity: easy (no external load for the first eight weeks), intermediate (increased external burden for the previous level from the 17th to the 24th week), and advanced (application of external load: elastic bands, shin guards, and free weights, from the 9th to the 16th week). Each month, a different type of session was developed. However, despite prescribing a structured training program with progressions for all participants, we consistently considered each individual’s unique progression. Recognizing that the participants had different levels of initial physical fitness, we tailored the plan to suit each person’s capabilities, ensuring that the exercises were accordingly adjusted to match their individual needs and optimize their development throughout the intervention. Each session was divided into three phases: the initial phase (10 min), consisting of a 5 min walk followed by a joint warm-up; and the fundamental phase (25 min), where the patients worked on a corresponding circuit of exercises. This circuit consisted of 4 cycles, with eight exercises each (50 s on, 15 s off); and a return to calm (10 min), where muscle stretching was performed (Choi et al., 2021) (Figure 1).

**Figure 2:**
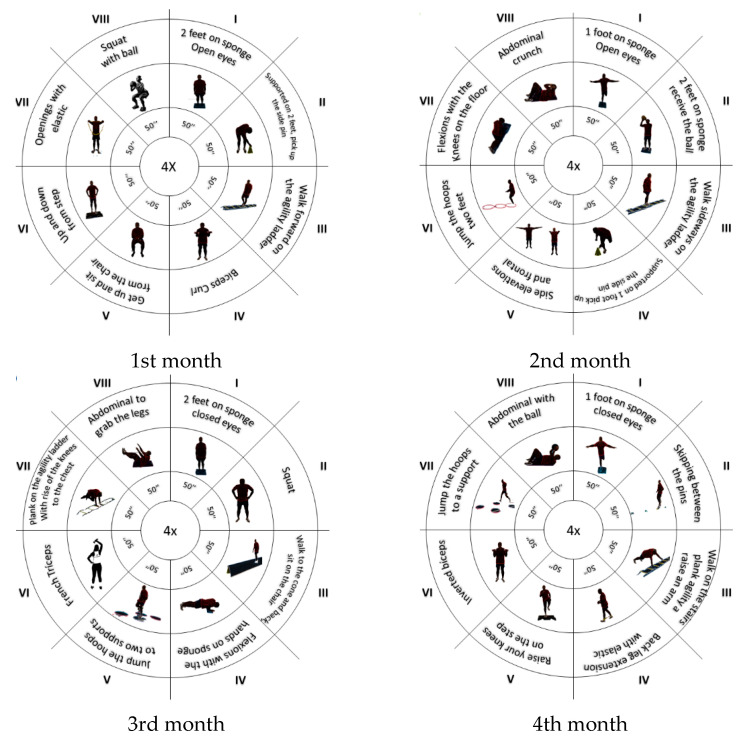

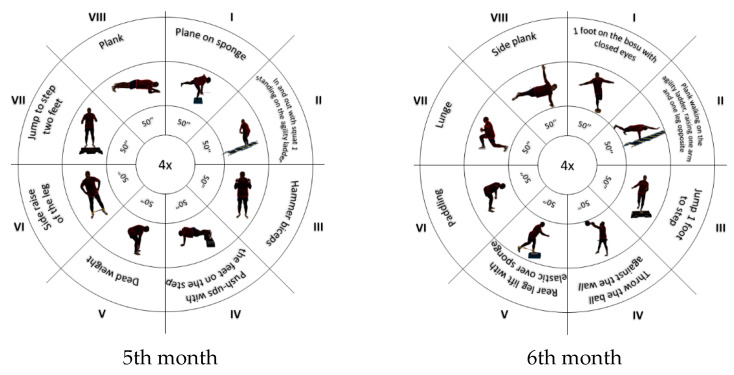
Intervention used for sensorimotor training.

Figure 2 shows the main exercises carried out during the intervention, with a short explanation of them. These exercises are part of a sensorimotor training program already applied to the population (Cabo et al., 2022), from which we obtained the results now published in this article. 

### 2.5. Measuring Instruments

A variety of instruments were used for the assessments under study. All measures were taken at the baseline and at the end of the intervention. Before the first measurement, all the participants were involved in a familiarization phase to adapt themselves to the different instruments and assessments associated with this project. The instruments included the following: a stadiometer (Seca 22, Hamburg, Germany); a scale; the Timed Up and Go test (TUG); a lower limb muscular endurance test; an upper limb strength test; the sit and reach test; and the behind the back reach test.

#### Evaluations

To assess the physical fitness of the participants, they wore tracksuit bottoms and were asked to remove accessories and any objects in their pockets. The following procedures were carried out:

1. Bodyweight and height. The participants were instructed to take off their heavy outerwear (coats, sweaters, etc.), shoes, and socks before the measurements. In addition, they were instructed to take off belts and other accessories (such as necklaces and bands) and to empty their pockets. A stadiometer (Seca 22, Hamburg, Germany) was used to measure their height. The measuring scale on this device was positioned perpendicular to the ground on a vertical surface. The participants were asked to stand with their arms relaxed along their bodies and their shoulders balanced. The height was measured in centimeters and rounded to the closest millimeter. A scale was used to measure their body weight. Weight (Kg)/height^2^ was the formula used to compute the BMI when the body weight was recorded in kilograms.
2. Agility and execution speed were assessed through the TUG test, which involved getting out of a chair, walking three meters in a straight line, going back, and then sitting down again, which was used to measure speed (Prasad et al., 2021).
3. Muscular endurance was evaluated using functional tasks such as rising from a chair or performing repeated bending and straightening movements for 30 s. These exercises targeted the lower limb strength and endurance, focusing on key muscle groups like the vastus medialis obliquus (VMO) and vastus lateralis (VL). The performance was quantified by reference to the number of repetitions completed within the 30 s, providing a measure of endurance and strength in the lower extremities, as outlined in Dunsky (2019).
4. The upper limb strength was assessed by counting the number of repetitions that a participant could perform in 30 s, using a specified weight during arm flexion–extension exercises. This measure provided a functional evaluation of the participants’ upper limb strength and endurance, emphasizing the capacity to sustain repetitive motion and muscular power. Additional details of the specific weight used and the positioning during the exercise would further clarify this methodology (Dunsky, 2019).
5. The lower limb flexibility was assessed using the “sit and reach” test, in which the participants gently bent over while sitting with one leg out in front of them, and then moved their hands down their leg till they touched (or passed) their toes (Mueller et al., 2021).
6. The upper limb flexibility was assessed using the “behind the back reach” test, which consisted of measuring with a ruler the distance between (or the overlap of) the middle fingers behind the back (Dunsky, 2019).

### 2.6. Statistical Analysis

To identify the normality of the sample, Kolgomorov–Smirmov’s test was considered, identifying a *p*-value higher than 0.05; therefore, normality was assumed (Field, 2013). In this line, Levene’s test was applied to assess the homogeneity of the sample. Parametric models were used to test the study’s hypotheses (O’Donoghue, 2012). Taking into account the CG and EG, a descriptive analysis of the sample was conducted to characterize the data using the mean and standard deviation (M ± SD).

Afterward, a Student’s T test was applied to compare the differences between the groups considering the first and second data collection (before and after the intervention). The effect size (ES) was calculated through Cohens *d*, and was considered trivial (0–0.2), small (0–2–0.6), moderate (0.6–1.2), large (1.2–2), very large (2–4), and extremally large (>4) (Hopkins et al., 2009). These metrics provide a clearer understanding of the magnitude of the observed changes between the pre- and post-intervention assessments. The Intraclass Correlation Coefficient (ICC) between the values obtained of each parameter’s reliability was determined as follows: <0.5 (poor), 0.5–0.75 (moderate), 0.75–0.90 (good), and ≥0.90 (excellent) (Koo & Li, 2016). In this line, the parameters of the present study showed values between 0.82 and 0.94, representing a good and excellent validity.

The statistical analyses were performed using Jamovi (Desktop version 2.5.2.0). Statistical significance was determined at *p* < 0.05.

## 3. Results

### 3.1. Descriptive and Inferential Analysis Considering the EG

Table 2 shows the main results according to the pre- and post-intervention values. All the analyzed variables showed improvement from pre- to post-intervention, particularly the “reach behind your back (right)” and “reach behind your back (left)”, variables which presented high values of ES.

### 3.2. Descriptive and Inferential Analysis Considering the CG

Considering the values of the CG, Table 3 shows no differences in any of the variables studied. It is identified that the variable with the lower *p*-values was “sitting and reaching” (*p* = 0.155). These values show that the CG did not improve their capacities compared to the intervention group.

### 3.3. Analysis of the Effect Size

According to the statistical analysis comparing the EG and CG, no significant differences were observed when comparing the group means.

The ES of the differences, along with their corresponding confidence intervals, are presented in Table 4. By analyzing the ES, we can gauge the practical significance of the intervention’s impact on various physical abilities, beyond mere statistical significance.

**Figure 3:**
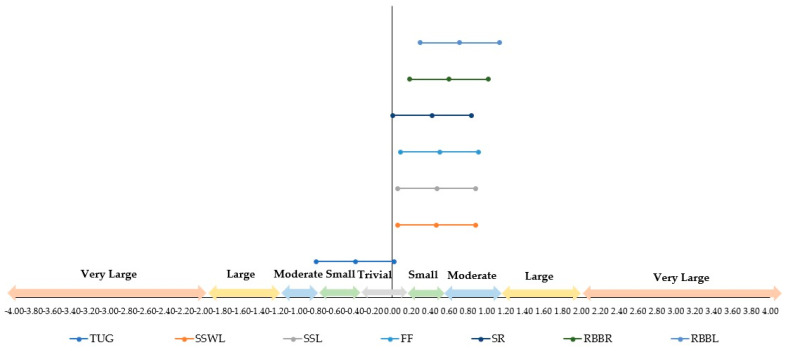
Interpretation of the effect size considering the analyzed variables. Note. Timed up and go (TUG); stand and sit with leaning (SSWL); stand and sit without leaning (SSL); forearm flexion (FF); sitting and reaching (SR); reach behind your back (right) (RBBR); reach behind your back (left) (RBBL). ES is considered trivial (0–0.2), small (0.2–0.6), medium (0.6–1.2), large (1.2–2), very large (2–4), and extremely large (>4) (Cohen’s *d*).

Figure 3 increases the comprehensive interpretation of the Table 4 data considering the proposal to interpret the ES values (Hopkins et al., 2009). This figure highlights that the TUG test had a negative and moderate ES. On the other hand, “reach behind your back (right)” and “reach behind your back (left)” were the variables that presented the largest positive ES. When we examine the distinctions between the groups, we observe once more that training influences the TUG test and the forearm flexion (Table 5).

### 3.4. Analysis of the MANOVA

According to the MANOVA test analysis, significant differences were observed in the TUG test, stand and sit without leaning, forearm flexion, and reach behind your back (left), with corresponding significance values of *p* < 0.001, *p* = 0.005, *p* = 0.003, and *p* < 0.001, respectively (Table 6).

When analyzing the F-values, we observed notable differences across the tests:

- TUG test: F = 79.0907, indicating a substantial variance between group means and a strong effect.
- Stand and sit without leaning and forearm flexion: F = 8.1732 and F = 9.4793, respectively, highlighting a more pronounced variance in this measure.
- Reach behind your back (left): F = 12.7230, showing a considerable difference between the groups.

These results reinforce the impact of the intervention on the key functional parameters. The notably high F-value in the TUG test suggests that the intervention had a strong effect on mobility performance, a critical factor in reducing the fall risk among the elderly. Additionally, the significant findings in the upper and lower limb mobility tests further support the relevance of sensorimotor training in improving the overall functional capacity.

## 4. Discussion

This study aimed to examine the effects of a sensorimotor training program on the physical capacities of older participants. The subjects shared similar characteristics in regard to age, weight, height, and BMI, which revealed that both the male and female groups were classified as overweight based on the values obtained.

Sensorimotor training uniquely integrates sensory and motor functions, emphasizing proprioceptive and neuromuscular coordination. This dual focus enables older adults to enhance their physical capacities such as strength, flexibility, and balance while addressing the multifaceted nature of mobility challenges (Freire & Seixas, 2024). Tasks involving balance and proprioception, such as standing on unstable surfaces or performing single-leg movements, train the body to respond dynamically to external stimuli. These exercises enhance both postural stability and neuromuscular control, equipping the participants with better reflexive and adaptive capabilities to prevent falls (Pšeničnik Sluga & Kozinc, 2024).

The discussion of tests assessing functional mobility and physical capacity in older adults can offer valuable insights into their effectiveness, sensitivity, and real-world application. The tests used—TUG, SSWL, SSL, FF, SR, RBBR, RBBL—each measure specific components of physical function, with relevance to the older population.

The timed standing and walking test, commonly represented by the TUG test, is widely utilized to assess balance, mobility, and the risk of falls in older adults. It involves standing up from a chair, walking a short distance (typically 3 m), turning around, and returning to sit down. This test is simple to administer and reflects real-life tasks, such as getting up from a chair and walking, which are fundamental for independence in daily activities. Research suggests that prolonged completion times are associated with an increased risk of falls and mobility impairments in older adults (Podsiadlo & Richardson, 1991). Furthermore, the TUG test is recommended by geriatric guidelines as a screening tool for fall risk (Barry et al., 2014). Based on the results of this study, the participants’ average time was 7.26 s, which decreased to 6.85 s following the intervention. These values align with the average found in other studies (Andrade et al., 2021; Bretan et al., 2013; B. P. Martinez et al., 2016).

Standing and sitting tests, particularly when performed on flat surfaces and inclined planes, assess lower body strength, balance, and coordination. These movements mimic activities of daily living, such as getting in and out of chairs or beds, and are indicative of an individual’s functional independence, challenging balance, and muscle control. The 30 s chair stand test (30s-CST), which measures how many times an individual can rise to a full standing position from sitting within 30 s, is commonly used to evaluate lower limb strength. It has been shown to have moderately high reliability and validity in community-dwelling older adults (Rikli & Jones, 1999). According to the reference values provided by Baptista and Sardinha (Baptista & Sardinha, 2005), the participants in the lower limb strength test ranked between the 75th and 90th percentiles. Our results indicate that the participants demonstrated excellent strength both initially and after the intervention, with noticeable improvements in the measured values. Additionally, since one key factor in enhancing lower limb proprioception is the learning effect, progressively increasing the difficulty of sensorimotor exercises contributes to improved proprioception.

The performance in these tests correlates with an individual’s ability to perform essential tasks, such as ascending stairs or rising from lower seating, which tend to decline with age due to reduced muscle mass and balance (Lord et al., 2002). The standing and sitting tests, both with and without inclination, offer a useful measure of functional ability and strength in different real-world conditions.

Forearm flexion, typically measured through the handgrip strength test, is a strong predictor of overall muscular strength and health status in older adults. It has been widely accepted as a marker for physical limitations and is associated with mortality, frailty, and disability in older populations. The handgrip test is practical, simple to perform, and provides an objective measure of upper body strength. It is particularly valuable in settings where comprehensive fitness tests are not feasible and serve as a standalone indicator of health and functional ability. Assuming the reference values of Baptista and Sardinha (Baptista & Sardinha, 2005), the participants in our study, and considering the lower limb strength test ranked between the 75th and 90th percentiles, their performance improved from the first assessment to the second.

Tests assessing flexibility and range of motion, such as sitting and reaching or reaching behind the back, are crucial for evaluating the upper body and spinal flexibility in older adults. These movements reflect the ability to perform daily tasks like dressing, grooming, or reaching for objects on shelves. As flexibility declines with age, so does the ability to perform these tasks comfortably and independently (La Greca et al., 2022). These tests help clinicians to assess the degree of joint and muscle stiffness and guide interventions aimed at improving flexibility and preventing further declines in functional capacity. The participants’ results were analyzed and compared with Ruivo (Ruivo, 2015), revealing that they fell between the 50th and 75th percentiles. In the upper limb flexibility test, however, the participants ranked between the 10th and 35th percentiles. Thus, contrasting their strength results, their flexibility scores were lower compared to the age-specific reference values. Nevertheless, the participants showed improvement in both tests following the intervention (Jones & Rikli, 2022). Together, these tests provide a comprehensive assessment of the critical physical abilities of older adults’ independence. The tests range from those that measure strength (e.g., handgrip, chair stands) and balance (e.g., TUG) to those that evaluate flexibility and mobility (e.g., reaching, sitting). The application of these tests in clinical and research settings supports the identification of declines in physical function, planning interventions, and tracking the progress of older adults. Each test offers valuable insights into the specific aspects of functional mobility, helping to tailor interventions that address the physical limitations associated with aging.

The analysis of the Control Group data in Table 3 reveals no statistically significant improvements across any of the measured variables, indicating that the physical capacities remained stable without intervention. The variable “sitting and reaching,” with a *p*-value of 0.155, showed the smallest difference, though it was still not statistically significant, suggesting minimal variation even in flexibility. These results underscore the effectiveness of targeted physical interventions, as the Control Group did not experience the gains observed in the intervention group. The findings highlight the critical role that structured exercise programs, such as sensorimotor training, play in enhancing balance, strength, and flexibility, which are essential for maintaining independence and reducing the fall risk in older adults. This study reaffirms that routine physical activity, especially interventions focused on balance and functional mobility, is crucial for preserving and improving the physical function as individuals age.

The results from the MANOVA analysis reinforce the effectiveness of sensorimotor training in improving functional mobility and motor control in the elderly. The significant differences observed in the TUG test (F = 79.0907, *p* < 0.001), stand and sit without leaning (F = 8.1732, *p* = 0.005), forearm flexion (F = 9.4793, *p* = 0.003), and reach behind your back (left) (F = 12.7130, *p* < 0.001) highlight the intervention’s impact on both dynamic balance and upper limb mobility. The high F-value for the TUG test suggests a particularly strong effect on functional mobility and fall risk reduction, which is crucial for elderly populations (Sherrington et al., 2017). These findings align with previous studies that indicate that sensorimotor training enhances postural control, strength, and flexibility, leading to improved functional independence and a reduced fall incidence (Granacher et al., 2011; Lesinski et al., 2015).

Furthermore, previous research has demonstrated that sensorimotor training improves proprioception and neuromuscular adaptation, which are essential for postural stability and fall prevention (Pizzigalli et al., 2011). The improvements in upper limb flexibility and strength, observed in the forearm flexion and reach behind your back tests, further support the idea that multimodal training interventions can enhance mobility beyond lower limb function (Muehlbauer et al., 2015).

These results reinforce the importance of structured sensorimotor programs in geriatric rehabilitation, particularly in individuals at the risk of mobility decline. Future studies should investigate the long-term retention effects and evaluate how different intensities and durations of training influence these functional outcomes.

The integration of balance-focused tasks in sensorimotor training has a direct impact on fall prevention. Improved proprioception and postural stability help older adults maintain control during perturbations. Training programs often simulate real-life scenarios, such as reaching for an object or recovering from a stumble, enabling the participants to develop reactive strategies that reduce fall risk (Pšeničnik Sluga & Kozinc, 2024).

Neuromuscular control is significantly enhanced through sensorimotor training, which emphasizes the synchronization of sensory input and motor output. Proprioceptive exercises, such as standing on unstable surfaces or performing single-leg tasks, strengthen neural pathways and improve the brain’s ability to process and respond to movement-related stimuli. This leads to faster and more accurate movement adjustments, improving overall coordination and reducing the likelihood of injuries (Aman et al., 2015).

Sensorimotor training integrates sensory input with motor actions to enhance physical performance and functional abilities. When we compare different groups that have undergone this training, distinct improvements are often observed in their performance on the TUG test and in forearm flexion. Overall, sensorimotor training offers comprehensive benefits by integrating sensory inputs and motor outputs. It significantly enhances functional mobility and strength, contributing to improved specific physical tasks. By focusing on the integration and improvement of sensory and motor pathways, individuals can achieve greater independence and quality of life (Gomiero et al., 2018; Kuş et al., 2023).

In this context, the application of the sensorimotor training program proved fundamental in enhancing the physical abilities assessed. The intervention demonstrated a significant positive impact on the participants’ QoL and their risk of falls. By addressing the key areas such as strength, balance, and mobility, the training not only improved their functional independence but also contributed to the prevention of fall-related injuries—a critical aspect of maintaining health and well-being in older adults.

One limitation of this study was the need to conduct the intervention during the COVID-19 pandemic, which required additional precautions to ensure safety. These included increasing the space between the participants, providing extra hygiene materials, and implementing physical distancing protocols. These measures may have influenced the nature of the training sessions by potentially reducing social interactions and the group dynamics, which are known to enhance the motivation and engagement in exercise programs. Additionally, the fear of contagion could have impacted the adherence to the training sessions, as some of the participants may have been hesitant to travel to the location or participate fully due to their concerns about exposure. This could have also introduced a selection bias, as more vulnerable individuals may have been excluded from the study, thereby limiting the generalizability of the findings to the broader older population. Future studies should consider these pandemic-related factors when interpreting the outcomes and designing interventions in similar contexts. Traveling to the location where the program was conducted also posed a challenge. In addition, the CG did not carry out any type of physical activity during the intervention. However, the lack of an active control condition (e.g., light stretching or participation in social activities) could lead to confounding factors such as placebo effects or differences in motivation between the groups.

In the future, it would be valuable to extend the study to include a wider range of age groups, including those who are still actively employed. This broader assessment would provide a more comprehensive understanding of the sensorimotor behavior across the lifespan, not just during aging. Early intervention could be implemented to improve the analyzed skills in this study, promoting healthier outcomes over time. In order to address the situation of lower flexibility test results in future programs, we recommend incorporating a dedicated flexibility training component with longer static stretches and dynamic stretching routines targeting the main joints and muscle groups. The use of individualized stretching protocols based on baseline flexibility assessments would better meet the specific needs of the participants.

## 5. Conclusions

The rising number of older adults underscores the need for tailored PA programs to support healthy aging. This study highlights the benefits of sensorimotor training, particularly in improving balance, strength, and overall physical function—key for reducing the fall risk and maintaining independence. While flexibility showed less improvement, balance and strength exercises effectively addressed broader physical needs. The intervention group demonstrated significant physical gains, unlike the Control Group, reinforcing the value of structured PA. Regular assessments of functional mobility are essential for adapting PA programs to ensure both effectiveness and practical applicability. Supporting an active lifestyle through such programs can help older individuals maintain autonomy, reduce the injury risk, and enhance the overall well-being.

The findings of this study provide valuable insights into the benefits of sensorimotor training for older adults, particularly in improving balance, coordination, and overall physical capacities. These results can be applied in real-world settings by integrating sensorimotor training into rehabilitation programs, fall-prevention strategies, and community-based exercise initiatives aimed at promoting functional independence in aging populations. Healthcare providers, physical therapists, and fitness professionals can incorporate sensorimotor training exercises, such as balance and proprioceptive training, into structured interventions to reduce fall risks and enhance mobility.

Despite its strengths, this study has some limitations. The sample size may limit the generalizability of the findings, and the intervention period may not fully capture the long-term effects. Additionally, individual variations in adherence and baseline physical condition could influence the outcomes. Future research should explore the long-term impact of sensorimotor training, investigate its effects on cognitive function, and examine its integration with other exercise modalities to maximize the benefits for older adults.

## Figures and Tables

**Table 1:** Characteristics of the CG and EG to contextualize the sample.

	N	Age (year)		Weight (kg)		Height (m)		BMI	
		Mean	SD	Mean	SD	Mean	SD	Mean	SD
Control Group	44	73.70	6.08	70.10	12.70	1.58	0.08	28.10	4.69
Experimental Group	46	72.40	6.88	68.40	14.20	1.58	0.09	27.40	5.03

Kg: kilograms; m: meters, BMI: body mass index; SD: standard deviation.

**Table 2:** Descriptive and inferential analysis of the pre- and post-intervention considering the EG.

Variables	Mean	SD	Student’s *t* Test	Mean Differences	*p*-Values
Timed up and go (pre) (s)	7.26	±1.23	3.90	0.416	<0.001
Timed up and go (post) (s)	6.85	±0.81			
Stand and sit with leaning (pre) (rep)	13.00	±2.30	−3.64	−1.043	<0.0001
Stand and sit with leaning (post) (rep)	14.00	±1.97			
Stand and sit without leaning (pre) (rep)	15.30	±2.95	−5.04	−1.370	<0.001
Stand and sit without leaning (post) (rep)	16.60	±2.57			
Forearm flexion (pre) (rep)	17.30	±5.83	−3.33	−2.522	0.002
Forearm flexion (post) (rep)	19.80	±4.04			
Sitting and reaching (pre) (rep)	−2.54	±8.70	−4.43	−3.565	<0.001
Sitting and reaching (post) (rep)	1.02	±8.24			
Reach behind your back (right) (pre) (m)	−13.80	±11.70	−7.29	−6.261	<0.001
Reach behind your back (right) (post) (m)	−7.50	±9.12			
Reach behind your back (left) (pre) (m)	−18.50	±11.00	−8.97	−7.391	<0.001
Reach behind your back (left) (post) (m)	−11.20	±9.43			

s: second; rep: repetition; m: meter; SD: standard deviation; *p* < 0.005.

**Table 3:** Descriptive and inferential analysis of the pre- and post-values considering the CG.

Variables	Mean	SD	Student’s *t* Test	Mean Differences	*p*-Values
Timed up and go (pre) (s)	8.15	±2.89	0.763	0.076	0.450
Timed up and go (post) (s)	8.08	±2.94			
Stand and sit with leaning (pre) (rep)	13.50	±3.42	−0.947	−0.318	0.349
Stand and sit with leaning (post) (rep)	13.80	±2.96			
Stand and sit without leaning (pre) (rep)	15.50	±4.31	−0.120	−0.045	0.905
Stand and sit without leaning (post) (rep)	15.60	±3.63			
Forearm flexion (pre) (rep)	18.30	±4.69	1.007	0.386	0.319
Forearm flexion (post) (rep)	17.90	±4.11			
Sitting and reaching (pre) (rep)	−0.61	±9.97	1.446	1.204	0.155
Sitting and reaching (post) (rep)	−1.82	±9.37			
Reach behind your back (right) (pre) (m)	−9.32	±12.50	1.100	2.636	0.277
Reach behind your back (right) (post) (m)	−12.00	±20.40			
Reach behind your back (left) (pre) (m)	−15.80	±12.90	−1.310	−1.056	0.197
Reach behind your back (left) (post) (m)	−14.80	±11.90			

s: second; rep: repetition; m: meter; SD: standard deviation; *p* < 0.005.

**Table 4:** Analysis of the effect size considering the pre- and post-intervention.

Variables	Pre-Intervention			Post-Intervention			ES	*σ*	95% CI	
	M	SD	*n*	M	SD	*n*			LL	UL
Times up and go	7.26	1.23	46	6.85	0.81	46	−0.39	0.210524737	−0.81	0.02
Stand and sit with leaning	13.00	2.3	46	14.00	1.97	46	0.47	0.211337371	0.05	0.88
Stand and sit without leaning	15.30	2.95	46	16.60	2.57	46	0.47	0.211372433	0.06	0.88
Forearm flexion	17.30	5.83	46	19.80	4.04	46	0.50	0.211727587	0.08	0.91
Sitting and reaching	−2.54	8.7	46	1.02	8.24	46	0.42	0.210802398	0.01	0.83
Reach behind your back (right)	−13.80	11.7	46	−7.50	9.12	46	0.60	0.213163448	0.18	1.02
Reach behind your back (left)	−18.50	11	46	−11.20	9.43	46	0.71	0.215029136	0.29	1.13

M: mean; SD: standard deviation; ES: effect size; CI: confidence interval; LL: lower limit; UL: upper limit; *p* < 0.005.

**Table 5:** Analysis of post-intervention values according to the intervention effect.

	Pre-Intervention		Post-Intervention			
Variables	Mean	SD	Mean	SD	Pre-Intervention *p*-Value	Post-Intervention *p*-Value
Timed up and go (pre) (s)	7.26	±1.23	8.15	±2.89	0.059	0.008
Timed up and go (post) (s)	6.85	±0.81	8.08	±2.94		
Stand and sit with leaning (pre) (rep)	13.00	±2.30	13.50	±3.42	0.395	0.702
Stand and sit with leaning (post) (rep)	14.00	±1.97	13.80	±2.96		
Stand and sit without leaning (pre) (rep)	15.30	±2.95	15.50	±4.31	0.737	0.111
Stand and sit without leaning (post) (rep)	16.60	±2.57	15.60	±3.63		
Forearm flexion (pre) (rep)	17.30	±5.83	18.30	±4.69	0.389	0.026
Forearm flexion (post) (rep)	19.80	±4.04	17.90	±4.11		
Sitting and reaching (pre) (rep)	−2.54	±8.70	−0.61	±9.97	0.330	0.130
Sitting and reaching (post) (rep)	1.02	±8.24	−1.82	±9.37		
Reach behind your back (right) (pre) (m)	−13.80	±11.70	−9.32	±12.50	0.086	0.182
Reach behind your back (right) (post) (m)	−7.50	±9.12	−12.00	±20.40		
Reach behind your back (left) (pre) (m)	−18.50	±11.00	−15.80	±12.90	0.285	0.113
Reach behind your back (left) (post) (m)	−11.20	±9.43	−14.80	±11.90		

SD: standard deviation; *p* < 0.005.

**Table 6:** Analysis of post-intervention values according to the MANOVA.

Variables	Sum of Squares	Mean Square	F-Values	*p*-Values
Timed up and go (post) (s)	6.85	±0.81	79.0907	** <0.001 **
Stand and sit with leaning (post) (rep)	14.00	±1.97	0.3351	0.564
Stand and sit without leaning (post) (rep)	16.60	±2.57	8.1732	** 0.005 **
Forearm flexion (post) (rep)	19.80	±4.04	9.4793	** 0.003 **
Sitting and reaching (post) (rep)	1.02	±8.24	7.1769	0.009
Reach behind your back (right) (post) (m)	−7.50	±9.12	3.7447	0.056
Reach behind your back (left) (post) (m)	−11.20	±9.43	12.7130	** <0.001 **

F-value interpretation intervals: F < 1 → no significant difference between groups; 1 ≤ F < 5 → small variation between groups (small effect); 5 ≤ F < 10 → moderate difference between groups (medium effect); F ≥ 10 → considerable difference between groups (large effect); F ≫ 10 → very large variation between groups, indicating a very strong effect; *p* < 0.005.

## Data Availability

Data are contained within the article.
